# Emission control of longitudinal optical phonon resonant thermal terahertz radiation from two-dimensional surface metal micro-structures on GaAs

**DOI:** 10.1080/14686996.2026.2724893

**Published:** 2026-09-21

**Authors:** Takumi Oshima, Daiki Yoshikawa, Yoshihiro Ishitani

**Affiliations:** Graduate School of Science and Engineering, Chiba University, Chiba, Japan

**Keywords:** Longitudinal optical phonon, radiation emission by phonon, surface structures in the region keeping LO phonon coherence, polarization free by two dimendional structures, control of emission and exitinction by selecting the size of 2D emission window

## Abstract

Thermal emission of radiation resonant with the longitudinal optical (LO) phonon (LORE) has been studied using surface metal line-and-space structures on semiconductors. The emission perpendicular to the surface and the independence of the peak photon energy on emission direction are significant features, unlike those of surface phonon polaritons (SPhP). However, this emission is restricted to the optical polarization perpendicular to the stripes. In this research, two-dimensional (2-D) square-lattice and ring-type surface structures are fabricated and almost unpolarized LORE spectra are observed at 8.5 THz at 630 K, persisting to 323 K. Although the local spectrum minima due to optical absorption via SPhP show dependence on structural periods, the LORE peak energy is found to be robust. The LORE intensity averaged over polarizations is nearly twice that of the one-dimensional structure. In contrast, when fabricating a rectangular emission window with the side length short enough for the LO-phonon coherence, no LORE is observed. A finite-difference time-domain calculation suggests that this phenomenon is due to the disappearance of electric dipoles. Thus, two-dimensional structures enable control over the allowance and prohibition of THz wave emission, as well as its polarization.

## IMPACT STATEMENT

Polarization-independent continuous-wave terahertz emission resonant with longitudinal optical phonons, using two-dimensional surface-metal microstructures on a GaAs wafer gives a promising mechanism for thermally stable THz emitters.

## GRAPHICAL ABSTRACT

**Figure:**
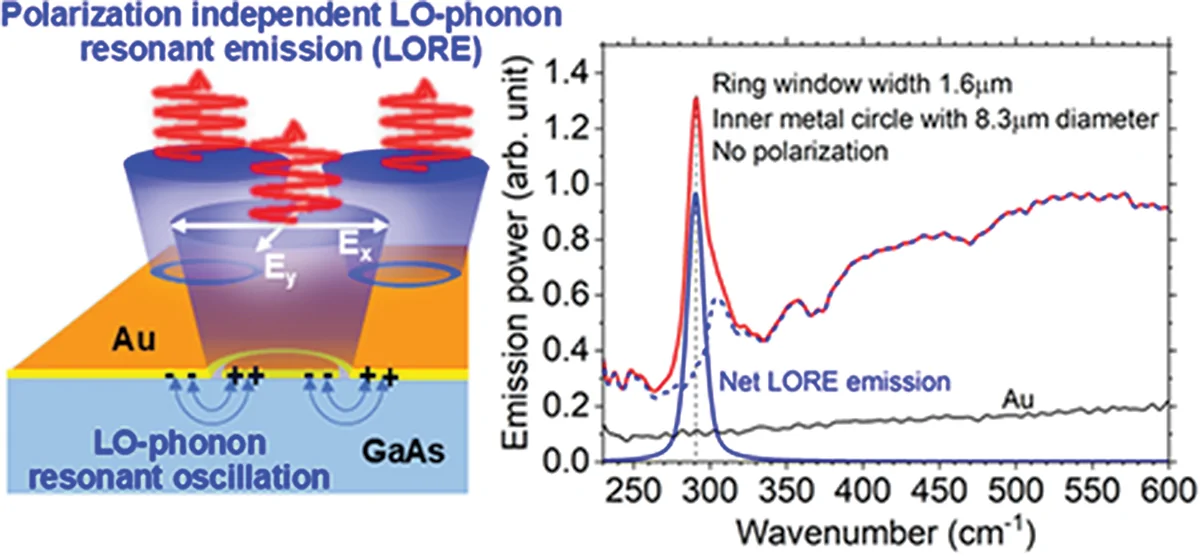

## 1. Introduction

The radiation energy range of 6.5–12 THz is referred to as the terahertz (THz) gap, where no compact continuous-wave (CW) laser device is available, except those based on frequency-difference techniques using nonlinear optical crystals [1–6]. In this energy region, quantum cascade lasers (QCLs) are now under development. However, radiation emission in the THz-gap is suppressed by the absorption and emission of longitudinal optical (LO) phonons. The interaction with phonons significantly broadens the electronic energy states, and consequently, the electronic inter-subband transition (ISBT) energy is transformed into LO phonons at a rate of 10^12^ s^−1^ or higher [7,8]. This rapid energy transfer to LO phonons is a significant barrier to radiation emission from the electronic system in the THz gap region when using the AlGaAs system, a popular high-crystal-quality material for QCLs. Recently, THz-wave emission via differential frequency generation using two high-frequency QCLs and a nonlinear crystal has been reported, with an output power of 200 μW at 2 THz [6]. However, there appears to be a significant barrier to achieving high-efficiency, high-output THz wave emission.

The interaction energy width of radiation and an electronic system is tens of meV at room temperature for bulk materials, which contrasts with values of less than 1 meV for the interaction of radiation with LO phonons, as demonstrated by infrared and Raman spectroscopy [9,10]. The technique of photonic crystals enables wavelength selection and thus narrows the emission spectra, while a sharp control of two- or three-dimensional nano- or microscopic surface structures is indispensable. Inoue *et al*. reported a significant reduction in the emission peak width to approximately 7 cm^−1^ at 1090 cm^−1^ for the spontaneous emission originating from an ISBT [11].

LO-phonon resonant radiation emissions (LOREs) were obtained as a pulse train based on the coherent phonon generation by the charge carrier drift in the surface electric field, such as photo-Dember effect or carrier generation in the anisotropic density of states using femtosecond lasers [12–16]. In contrast, we have reported continuous wave (CW) LORE in the 8.5–21 THz region using undoped (u-) semiconductor wafers at 430–630 K by generating surface electric dipoles in line-and-space microstructures of metal plates (metal stripes) on the wafers [17–22]. No electronic excitation is involved in this radiation emission process. This thermal radiation emission was found to originate from the LO-phonon resonant vibration of pairs of positive and negative charges formed at pairs of metal/semiconductor interfaces, as illustrated in Figure 1. This LORE is allowed in all directions, including the surface normal direction, and has a constant emission peak energy independent of the emission direction. These properties contrast sharply with those of emissions based on surface phonon polaritons (SPhPs), showing a significant polar-angle dependence of the emission wavelength and the prohibition of emission to the surface normal direction without controlling the surface grating period [23–27], i.e. a sharp control of one- or two-dimensional periodic structure is required. In many cases, the SPhP emission peak is observed within the Reststrahlen band, surrounded by a high-intensity blackbody-like background [26,27]. In contrast, covering the surface with a metal, such as Au, in the LORE structure reduces the emissivity in the metal-covered region. For wide-bandgap materials with wide Reststrahlen bands between the LO and transverse optical (TO) phonon energies, the emission peak is shifted to a lower energy from the original LO phonon energy. This phenomenon is due to the modulation of the dielectric function near the surface [20]. Furthermore, the emission peak energy depends on electron density for direct electronic transition-type materials, but not for indirect transition-type materials [21]. The emission frequency from the u-GaAs window is stable at 8.5 THz at 630 K and independent of the window width in the range of 1–10 μm [18].

**Figure 1.**
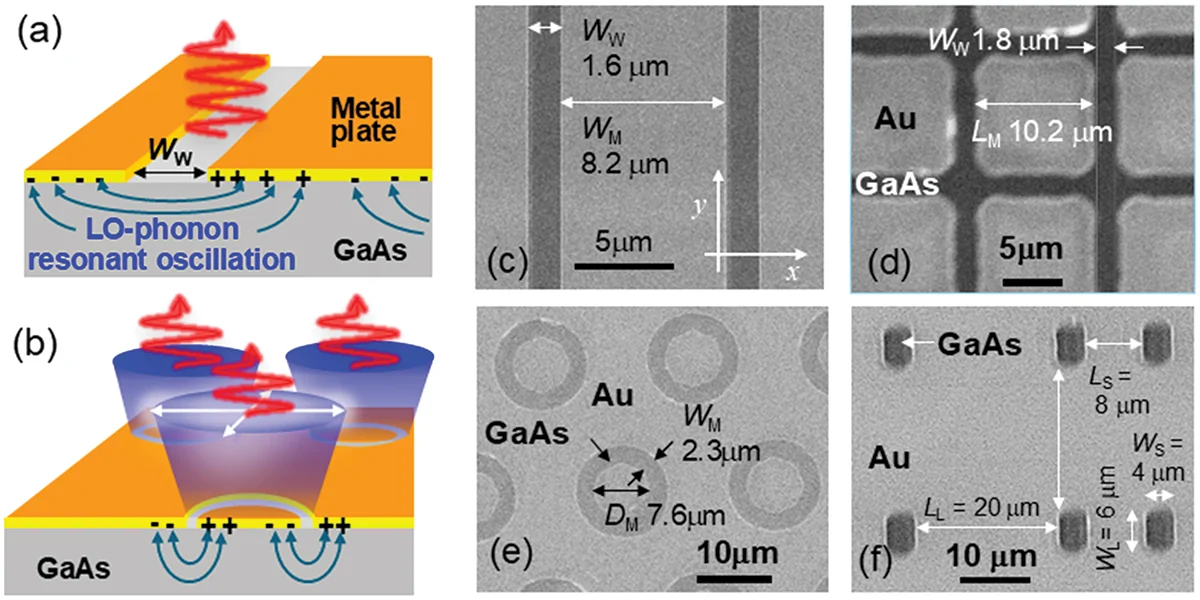
Mechanism of LO phonon resonant radiation emission (a) and (b) and examples of SEM images of GaAs–Au surface structures: (c) stripes, (d) square-lattices, (e) rings, and (f) square-windows.

Despite these advantageous features of the LORE, the emission based on one-dimensional (1-D) metal stripe structures is restricted to optical polarization perpendicular to the stripe lines (E_⊥_) [17–22]. The enhancement of emission, extension of polarization, and suppression of blackbody-like background emission are key issues for future improvement. We have reported a reduction in the background emission by narrowing the emission window width, while the polarization selection remains. It is thought that two-dimensional (2-D) structures offer a solution to this issue. However, we have no fundamental information on the emission property of 2-D structures.

In this report, we investigate the LORE properties of 2-D surface structures. The relaxation of polarization selection would lead to higher emission intensity. Understanding how geometrical factors in 2-D structures affect radiation-emission efficiency is critical to the application of this emission mechanism. The suppression of the blackbody-like background emission is also a viewpoint of the investigation. When we apply this radiation emission to information communication, the on-off switching of the radiation is a key technology. A finding of controllable surface structures that enable the switching of emission allowance and prohibition would pave the way for communication applications. We extend the discussion to a characteristic feature of emission prohibition in short-stripe windows. The discussions are based on the observed properties of the emission from 1-D stripe structures. We investigate more detailed LORE properties of 1-D structures than before, including diffraction, effective medium, wire-grid polarizer, and LO-phonon resonant electric dipole, which were not fully clarified in the previous reports. The emission properties of 2-D structures are discussed on the basis of those of 1-D structures.

## 2. Experimental section

Au films with a thickness of 55–75 nm, which is thicker than the estimated skin depth of 25 nm at the LO-phonon resonant photon energy, were deposited by a magnetron sputtering method (LAS2020, LABOTEC, Japan) on patterned masks on u-GaAs wafers via photolithography after surface cleaning by UV-ozone exposure. Four types of surface patterns were fabricated, type A (1-D stripe): Au line-and-space structures, with the emission window width *W*_W_ and metal width of *W*_M_, type B (square lattices) with square metal regions with a side length *L*_M_ and the GaAs window width of *W*_W_, type C (ring): concentric circular structures with an inner circle diameter of *D*_M_ and an emission window width of *W*_W_, and type D (square window): rectangular emission window structure with the side lengths of *W*_L_ and *W*_S_ (*W*_L_ > *W*_S_). *L*_S_ and *L*_L_ are the shorter and longer distances between window regions. The sample structures are summarized in Table 1. Examples of scanning electron microscope (SEM) images are shown in Figure 1.

**Table 1.** Structural properties of samples. The structural parameters of *W*_W_, *W*_M_, *L*_M_, *D*_M_, *L*_S_, and *L*_L_ are denoted in Figure 1.

Type	Structural feature	Size details
Bare	Without metal u-GaAs, n-GaAs	–
A	Line and space	(*W*_W_, *W*_M_): (1.6, 8.4), (2.0, 8.0), (6.0, 8.0), (10, 10), (17, 17)
B	Square lattices	(*W*_W_, *L*_M_): (1.8, 10.2)
C	Concentric circles (donut)	(*W*_W_, *D*_M_): (2.3, 7.6), (1.6, 8.3), (2.0, 26)
D	Rectangular squares	(*W*_s_, *W*_L_, *L*_S_, *L*_L_): (2, 20, 2.5, 14), (2, 10, 2.514), (4, 6, 8, 20)

Reflectance and emission spectra were obtained using a Fourier-transform infrared spectrometer (FT-IR 660 plus, JASCO, Japan), using a DLTGS detector with a KRS-5 window for 400–5000 cm^−1^ or a Polyethylene window for 200–600 cm^−1^. KBR or Mylar beamsplitters were used for the respective energy regions. The setup of the measurement system is exhibited in Figure 2. The energy resolution was approximately 1 cm^−1^. In emission spectrum measurements, dried (dew point of −70°C) and decarbonized air from a purge-gas generator (75–72JA100, Parker Haniffin, United States) was flown into the sample chamber at a constant rate. Samples were heated using an Ohmic heater, and their surface temperature was measured with a chromel-alumel thermocouple. The temperature was maintained at 630 K or 323 K during the emission spectrum measurements. The observation spot diameter was 2 mm. A polarization component was selected by a wire-grid KRS-5 polarizer. Emitted radiation was collected by an off-axis parabolic Au-coated mirror with a diameter of 50 mm and a reflected focal length of 157 mm. In the reflectance measurements, light from a globar was focused on a sample using an off-axis parabolic Au-coated mirror with a 50-mm diameter and a 120-mm reflected focal length, and the reflected light was focused on a detector.

**Figure 2.**
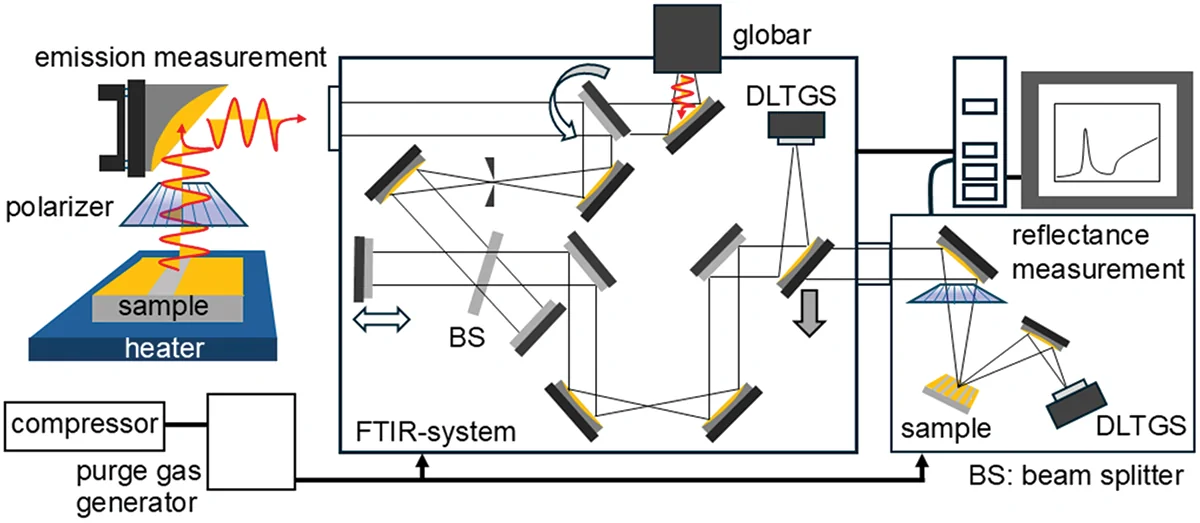
Overview of the IR emission and reflectance measurement system setup.

## 3. Results and discussions

### 3.1. Emission from 1D line-and-space structures and the emission mechanism

In this section, we extend the perspective on the emission properties of the 1-D stripe structures from the previously reported results [18,22]. The structure details are exhibited in Table 1. The SEM image is shown in Figure 1. The dependence of the LORE intensity on stripe structures is discussed by comparing it to that of bare GaAs. In particular, the spectrum is decomposed into the LORE and background emission components, accounting for the radiation selection due to the wire-grid polarizer of the metal gratings. This analysis serves as the basis of the discussion on the emission properties of the following 2-D structures. The emission efficiencies are compared with those of the radiation from u-GaAs and n-GaAs wafers without metal stripes, which are denoted as bare GaAs wafers in this paper.

Figure 3(a,b) shows the emission spectra of the bare u-GaAs wafer and the A-type samples with (*W*_W_, *W*_M_) = (17, 17), (10, 10), (6.0, 8.0), (2.0, 8.0), and (1.6, 8.4). The structural parameters of the emission window width *W*_W_ and the metal width *W*_M_ are denoted in Figure 1. When *W*_W_ is 17 μm, the emission spectrum is similar to that of bare u-GaAs. The intensity of this sample is consistent with the average of those of Au and bare GaAs. This emission is not polarized. The directions of *x* and *y* are defined in Figure 1(a). In contrast, each emission from the structures with *W*_W_ ≤ 10 μm has a peak at 285 cm^−1^, resonating with the LO phonon at 630 K, only for the E_x_-polarization. Figure 3 shows an image of the electric field distribution calculated by a finite-difference time-domain (FDTD) method at 291 cm^−1^, assuming room temperature. In this simulation, the Yee algorithm was adopted [20,21]. The space and time resolutions were expressed as λ/250 and λ/(250*c*), respectively, using wavelength λ and the velocity of light *c*. The calculation area was surrounded by an absorption region with a width of 0.6λ, suppressing the optical reflectivity at the boundary to 10^−5^. In this simulation, a wave-packet with eight cycles of a sinusoidal wave was incident on the surface structure from the front side, and the plotted electric field pattern is at 0.3 ps after the last cycle of the incident wave has passed the surface structures. The emission spectrum, integrated over the time region, excluding the reflectance component, is plotted. The high emission intensity and this electric field pattern near the emission window are observed in the vicinity of the LO-phonon energy. The adopted physical constants of the TO phonon energy, LO phonon energy, and static dielectric constant are ω_TO_ = 266.8 cm^−1^ [28], ω_LO_ = 291.8 cm^−1^ [28], and ε(0) = 13.18 ε_0_ [29], respectively. ε_0_ is the dielectric constant in vacuum. The dielectric constant at the high frequency limit ε(∞) = 11.0 ε_0_ is obtained from the Lyddane-Sachs-Teller relation. Numerical simulations were conducted using physical constants at room temperature, as not all constants at high temperatures are well established. The following equation gives the dielectric function used in this simulation

(1) $$\varepsilon \left(\omega \right)=\varepsilon \left(\infty \right)+\frac{\left(\varepsilon \left(0\right)-\varepsilon \left(\infty \right)\right)\omega_{T}^{2}}{\omega_{TO}^{2}-\omega^{2}+i\gamma_{T}\omega }.$$

**Figure 3.**
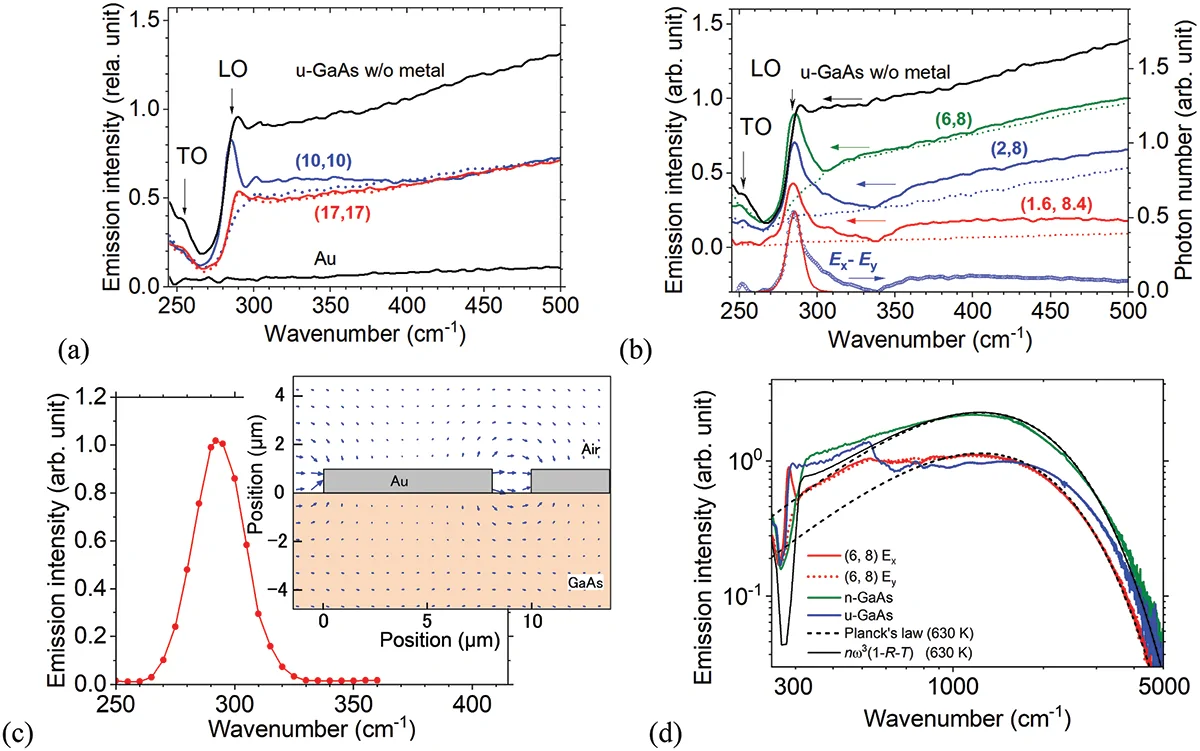
Emission spectra for type A: (a) GaAs without metal stripes and (*W*_W_, *W*_M_) = (17 μm, 17 μm) and (10 μm, 10 μm), (b) (*W*_W_, *W*_M_) = (1.6 μm, 8.4 μm), (2.0 μm, 8.0 μm), and (6.0 μm, 8.0 μm), (c) electric field image in a cross section and emission spectrum calculated by FDTD method, and (d) emission spectra in a wide photon energy region. The energies of the TO and LO phonons are denoted by arrows in (b). The dotted and solid curves in the spectra shown exhibit the polarization components of *E*_x_ (*E*_⊥_) and *E*_y_ (parallel to the stripes: E_//_), respectively. The spectra are plotted on a relatively comparable intensity scale and do not show the intensities per window line. The electric field image is obtained at 291 cm^−1^, consistent with the LO-phonon frequency at the simulation temperature. The simulated spectrum shows the time-integrated emission intensity.

Here, γ_T_ is the damping factor of the TO phonon. The ideal conductance is adopted for the metal. This figure shows the formation of electric dipoles resonating with the LO phonon in the surface region. This type of electric-field oscillation was also observed in other energy regions, while the intensity vanishes as the photon energy moves away from the resonance. It was reported that the LO-phonon energy decreases as the temperature increases, and the extension of the reference data [30] gives the value of 285 cm^−1^ at 630 K. Thus, the experimentally observed peak at 285 cm^−1^ has been ascribed to emission from electric dipoles yielded by coherently vibrating pairs of positive and negative charges formed on pairs of Au/GaAs/Au interfaces [18]. It is thought that the coherence extends into the window region up to 10 µm in width. The low emission intensity in the Reststrahlen band, 260–285 cm^−1^, is attributed to the negative real part of the dielectric function of u-GaAs in this region.

Each spectrum was decomposed into two components: the LORE and the background thermal emission, which was affected by several factors, including absorption that generates SPhPs, a wire-grid polarizer, and diffraction due to the narrow window width. In the present discussion, we have extracted the operating factors for thermal emission in surface GaAs-metal microstructures by also investigating the background blackbody-like emission for E_x_ and E_y_ polarizations.

Figure 3 shows the spectra over a broader range of photon energy. The dashed curves are the spectrum subject to Planck’s law in arbitrary units. These curves are fitted to the experimental plots in the high-energy region above 1000 cm^−1^. The emission spectrum of the bare u-GaAs shows a significant intensity drop above the photon energy of 550 cm^−1^, while it approaches the intensity of the n-GaAs bare sample above 4000 cm^−1^. This spectrum feature of u-GaAs was attributed to the transmission of radiation; the spectrum feature was reproduced by the function of *n·*ω^2^*·*(1-*R-T*), where *n*, ω, *R*, and *T* are the Bose-Einstein factor, the photon energy, reflectance, and transmittance, respectively [19]. However, some factors affecting the spectrum shape remain veiled. In contrast, the emission spectrum of a bare n-GaAs wafer exhibits two features: no drastic spectrum drop above 550 cm^−1^ and slightly higher intensity than that of the u-GaAs sample in the region of 320–500 cm^−1^. This higher intensity of the n-GaAs can be attributed to the higher absorbance and emittance due to the plasmon in the n-doped wafer. The spectrum edge at 320 cm^−1^ is higher than the LO phonon energy, which is attributed to the LO phonon–plasmon coupling (LOPC) [21]. Based on these spectral features of the bare wafers, we discuss the details of the background emission properties of the A-type samples in the following.

The spectrum for the A-type (6.0, 8.0) structure on the u-GaAs wafer shows a lesser decrease in intensity above 500 cm^−1^ than that of the bare u-GaAs. This feature is attributed to the greater absorbance and emittance in the Au-GaAs composite than in u-GaAs or Au. In contrast, in the photon energy region above 1000 cm^−1^, the intensity is thought to be dominated by the average of the GaAs emission window and Au plates.

In the previous paper, the cooperation of several factors of diffraction, wire-grid, effective medium, and SPhP, depending on structural property, was unsolved. The variation of the intensity of the background blackbody-like emission is more significant than that of the LORE intensity. The details of the LORE intensity based on the electric-dipole emission have been discussed in a previous paper [19]. In contrast, the blackbody-like emission intensity is investigated in this paper, which serves as the basis for the discussion of 2-D structures.

The emission intensities of the samples of (17, 17) and (10, 10) at 500 cm^−1^ agree with the sum of the 50% fractions of the intensities of the u-GaAs wafer and the Au film. In contrast, both the E_x_ and E_y_ intensities of the (6.0, 8.0) sample are higher than the sum of 6/14 of the intensity of the GaAs wafer and 8/14 of that of the Au film. The reduction of the intensity of the E_y_ component is minor for this sample. This property suggests that the emissivity of the effective medium, reflecting a higher contribution from GaAs, dominates for *W*_W_ = 6.0 μm. When reducing the share of GaAs window area, the intensities of both polarizations reduce. This feature suggests that the reduction in radiation emission due to diffraction, driven by the narrow window width, dominates for *W*_W_ = 2.0 μm and narrower. At the same time, the wire-grid structure selects the E_x_-component.

Figure 4 shows a spectrum of the A-type (2.0, 8.0) sample at 323 K. The spectrum peak at room temperature was too weak to be analyzed. This is attributed to thermal noise from the mirrors before the interferometer, and the significant reduction in emission intensity is proportional to *n*^*m*^, where *n* and *m* are the Bose-Einstein factor and a real number, respectively. *m* was found to be 2.2 by considering the intensity at 630 K. This value depends on material and stripe structure and was found to be 2.5 for a GaAs sample (430–580 K) [19]. 323 K is considered close to the lower limit of measurement temperature in our system. In this spectrum, the emission peak is located at 292 cm^−1^, which is close to the LO-phonon energy at room temperature.

**Figure 4.**
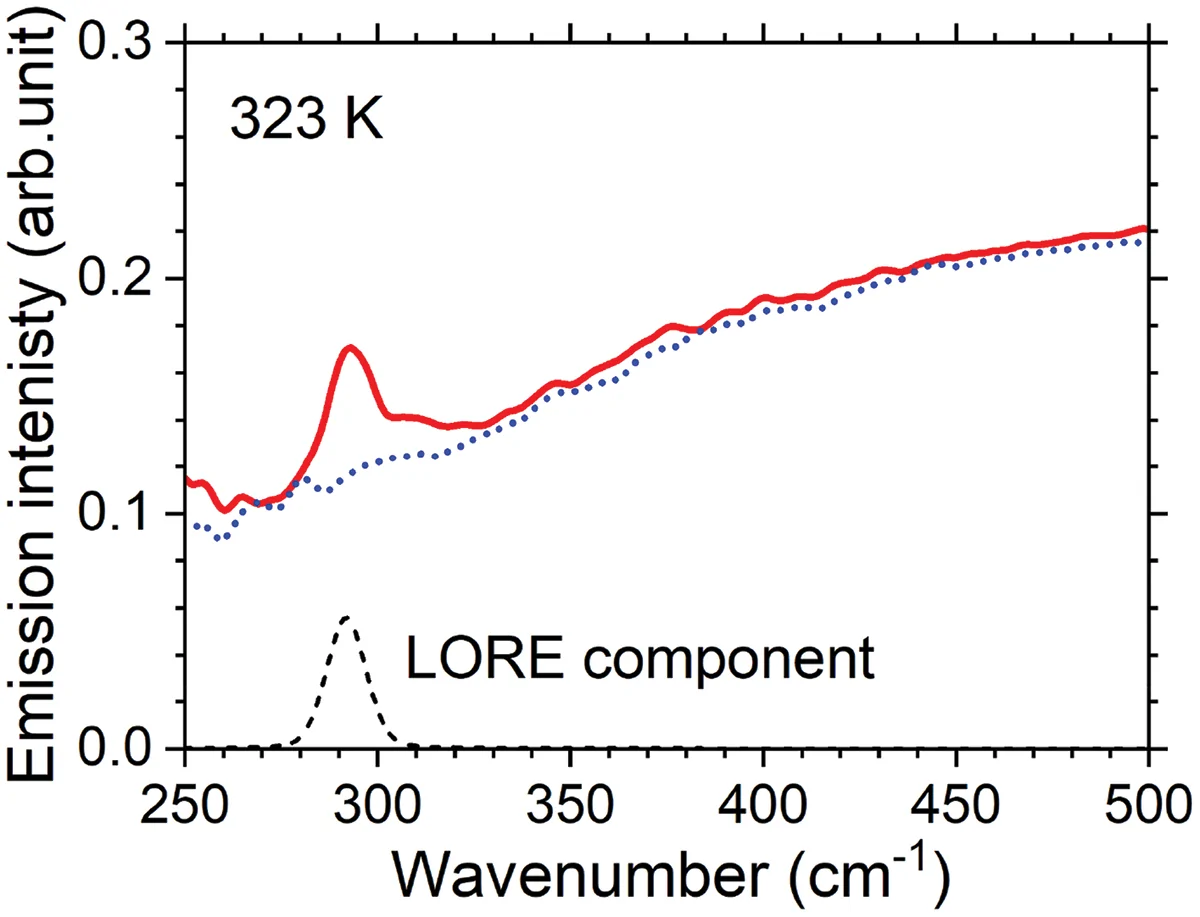
Emission spectrum for the A-type (2.0, 8.0) sample at 323 K. Solid and dotted curves show the polarization perpendicular and parallel to the stripes. The dashed line is the LORE component.

The diffraction effect becomes significant when *W*_W_ is reduced to 1.6 μm. The spectral features of the background emission in the 300–400 cm^−1^ range for the E_x_-component are affected by three factors: diffraction, absorption by SPhPs, and the selective transmission of radiation polarized perpendicularly by the wire-grid polarizer. It is thought that the operation of the wire-grid polarizer mitigates the diffraction-based decrease in the E_x_-emission intensity. At the same time, the diffraction effect provides another function, producing a range of radiation wavevectors within the emission window, allowing a range of SPhP energies. The (10, 10) structure shows a slightly higher intensity of the E_x_-polarization component and no intensity drop in the 300–380 cm^−1^ range. In this sample, the wire-grid effect is observed at a higher E_x_ intensity than E_y_ intensity in the range of 300–380 cm^−1^, but the diffraction effect, which allows absorption due to SPhPs, is negligible. This feature is contrasted with the property of the sample with (6.0, 8.0) and other samples with lower *W*_W_. The energy dispersion curve of SPhP shows several crossings with the light lines in the 300–400 cm^−1^ range. The broader-spectrum dip in this energy region is attributed to a more substantial diffraction effect at narrower window widths.

As indicated in section III-C and in previous papers [18,22], the blue line in Figure 5 shows the dispersion curve of SPhP calculated by the following equation [23].

(2) $$k_{//}=\pm \left(\frac{\omega }{c}\right)\sqrt{\frac{\varepsilon_{1}\varepsilon_{2}}{\varepsilon_{1}+\varepsilon_{2}}}+p\frac{2\pi }{L}.$$

**Figure 5.**
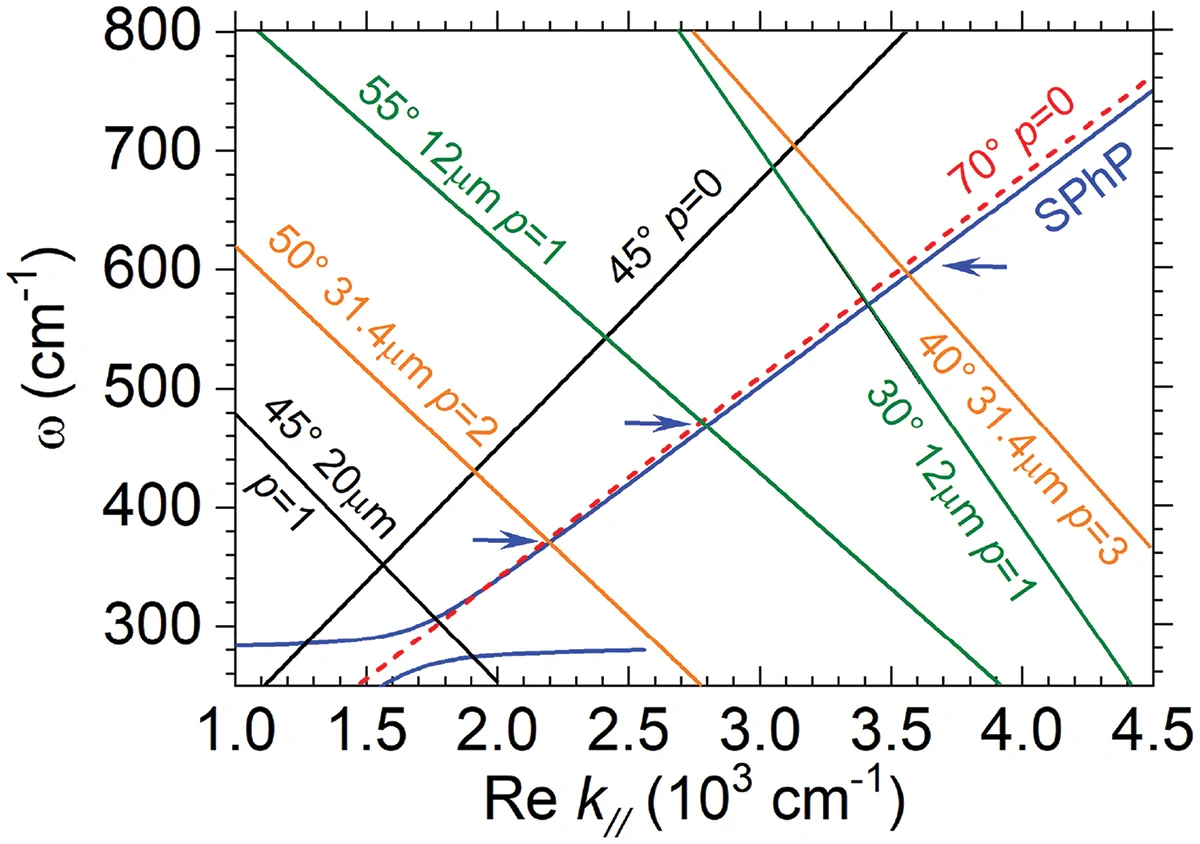
Dispersion curve of SPhP. The polar angle of the light wavevector θ, stripe period *L*, and *p* values are denoted for light lines.

Here, *k*_//_ is the *k* vector component parallel to the surface. ε_1_ and ε_2_ are the dielectric functions of the two layers forming an interface. *p* is an integer. *L* is the period of the surface structure. The dielectric constants at static and high-energy limits are the same as adopted in the FDTD calculation, but the TO mode energy of 258 cm^−1^ is utilized to give the LO mode energy of 285 cm^−1^ at 630 K. Although the dielectric function near the surface is modified by the electric dipole yielded by the surface microstructures, the deviation from the original dielectric function was found to be negligible [18]. The maximum photon energy of the local emission minimum for A-type samples, 360 cm^−1^, corresponds to the cross point of the SPhP dispersion curve and the light line with the polar angle θ = 70° and *p* = 0. Because the energy of the intersection of the light line and the dispersion curve of the higher energy branch increases with the increase in the θ of the *k* vector, the enhancement of diffraction allows the generation of SPhP with high *k*_//_/*k* and thus high SPhP energy. Thus, the high diffraction in the window region is consistent with the drop in the emission spectrum at 300–360 cm^−1^.

The E_x_ and E_y_ spectrum polarization components show intensity coincidence at a photon energy in the 300–350 cm^−1^ range for samples with *W*_W_ narrower than 10 μm, including those reported in previous papers [18]. This feature suggests that the component of the blackbody-like emission augmented by the wire-grid effect is completely absorbed at the minimum in the radiation intensity when *W*_W_ is 6 μm or lower. As the wavevector deviates from the value at this intensity minimum, the absorption due to SPhPs is weakened, allowing the emission from blackbody radiation generated in the region without any LO phonon. Thus, the remaining emission component at the intensity minimum is considered to be free from the formation of electric dipoles by the polarization charges at the metal/GaAs interfaces.

When we subtract the intensity of the E_y_-component from that of the E_x_-component, the resulting spectrum in 300–350 cm^−1^ comprises the LORE elements and blackbody-like emission via the wire-grid effect, but is reduced by SPhP formation and suppressed in the Reststrahlen band. Thus, the LORE components of the A-type samples are obtained via three processes: (1) subtraction of the E_//_ component from the E_⊥_ component, (2) fitting a trial peak function of a quai-Voigt function to the residual component in process (1), and finally (3) subtracting the adjusted peak function from the E_⊥_ component, yielding the background emission. The difference spectrum of E_x_- and E_y_-components presented by photon number was fitted by a quasi-Voigt function in the range below 291 cm^−1^, which is the sum of a Lorentzian and a Gaussian. The agreement between the experimental and theoretical functions indicates that this fit is valid up to 291 cm^−1^. The best-fit LORE spectrum for the sample with the (2.0, 8.0) structure, shown in Figure 3(b), has adjusted parameters of a peak energy of 285.5 (±0.1) cm^−1^, FWHM of 11.6 (±0.2) cm^−1^, and a Lorentzian fraction *r*_L_ = 0.44 (±0.15). This result suggests the inclusion of fluctuations of the emission peak energy or the effect of the refractive index profile. The resultant LORE and background blackbody-like emission affected by SPhP are plotted in Figure 6(a). This spontaneous emission with 11.6 (±0.2) cm^−1^ at 285.5 (±0.1) cm^−1^ is contrasted with the broader spontaneous emission via ISBT in QCL structures, e.g. an FWHM of 350 cm^−1^ for the peak at 1150 cm^−1^ [30]. At 323 K, the emission peak has an energy of 291.9 (±0.2) cm^−1^, FWHM of 12.0 (±0.2) cm^−1^, and *r*_L_ = 0.25 (±0.13). The intensity was found to be 9.6% of that at 630 K, giving the *m* value of 2.2 in *n*^*m*^.

**Figure 6.**
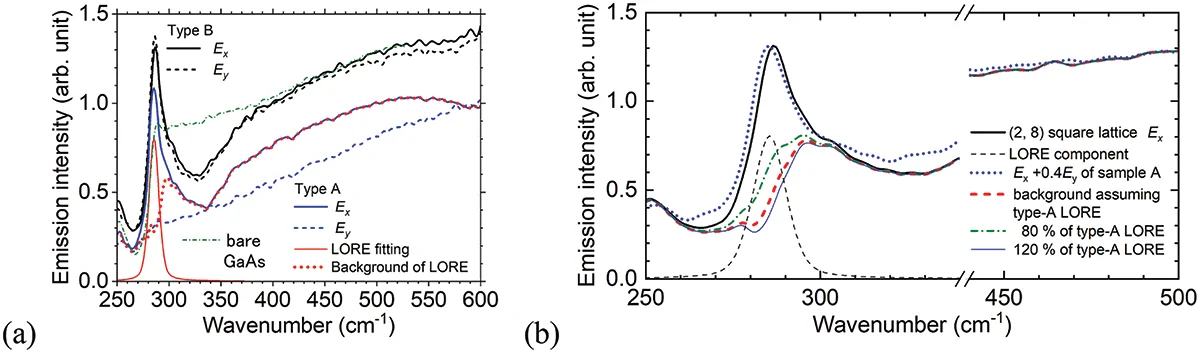
Emission spectra of type A and type B (1.8, 8.4) structures at 630 K, (a) spectrum comparison between these two samples and the spectrum decomposition for the A-type sample, (b) spectrum decomposition for the B-type sample.

The mixing of Lorentzian and Gaussian components can be attributed to the mechanism of forming surface electric dipoles, as reported in a reference on LORE in GaN [20]. In materials with a wide energy gap between the TO- and LO-phonon energies, the energy of the surface-localized LO phonon shifts to a lower energy, e.g. 35–50 cm^−1^ for GaN, and additional LO-like modes can be generated. This effect is considered weak for GaAs because of the lower LO-TO splitting energy of 25 cm^−1^ than 178 cm^−1^ of GaN. The peak energy 291.9 (±0.2) cm^−1^ at 323 K, shown in Figure 5, agrees with the estimated value of 292.3 cm^−1^ using 285 cm^−1^ at 630 K. Thus, the effect of the decrease in the effective LO mode, observed for GaN, can be neglected for GaAs. The reason for the inclusion of the Gaussian is not clear at present.

It was reported that the emission intensity depends on the window and metal widths. When *W*_W_ is 4 μm or greater, the emission intensity is determined by a trade-off between the increase in the electric dipole, resulting from the increase in *W*_W_ or the distance of pairs of positive and negative charges, and the increase in the annihilation rate of the surface-localized LO phonons, resulting from the spatial expansion of the electric flux connecting pairs of Au plates, over the LO-phonon coherence [18]. The dependence of the LORE intensity on the structural factors has been systematically investigated for various *W*_W_s in a previous report [19]. We have found that the LORE intensity has a high correlation with the volume occupied by electric flux connecting pairs of Au plates: *V*_C_ (supplemental material). When *V*_C_ is too high, LO phonons are considered to be scattered at a high rate; in contrast, small *V*_C_ has an advantage for the LO-phonon coherence, but the electric dipole is restricted because of the short distance of pairs of positive and negative charges. The variation of LORE intensity due to the 0.2 µm variation in *W*_W_ can be resolved.

### 3.2. Emission from square lattice structure

Figure 6(a) shows the spectrum comparison of the A-type (2.0, 8.0) and the B-type (1.8, 10.2) samples for the polarization components of E_x_ and E_y_. This figure includes the spectrum of the bare u-GaAs wafer. The emission intensity of u-GaAs is adjusted to match the E_x_-component of sample B at 500 cm^−1^. This spectrum is considered to show the upper limit of the blackbody-like emission of the E_x_-component without the absorption by SPhPs located in the vicinity of 330 cm^−1^. The spectra of the B-type sample show the disappearance of polarization anisotropy. In the following paragraph, we discuss the total emission intensity of the B-type sample by comparing it with that of the A-type sample. The decrease in the E_x_-emission intensity in the vicinity of 570 cm^−1^ can be attributed to the absorption by SPhP. The light line with θ = 30° and *p* = 1 shown in Figure 5 has an intersection with the dispersion curve at 570 cm^−1^.

The background emission with the E_x_-component of the B-type sample is enhanced compared to that of the A-type sample. This feature is ascribed to the emission from the window lines parallel to the *x*-axis. In the following, we discuss the LORE intensity of sample B by decomposing the spectrum into its LORE and background components.

For the spectrum of sample B, it is expected that the E_x_-component is the sum of the E_⊥_-component of the window lines parallel to the *y*-axis and E_//_ of the windows parallel to the *x*-axis, while it is difficult to obtain only the E_//_-component, contrasted with A-type samples. Starting from that viewpoint of the sum of the E_⊥_- and E_//_-components, we discuss the validity of the structural properties of the background emission spectrum obtained by subtracting the LORE component from the E_x_-spectrum, and the LORE intensity. Figure 6(b) shows the decomposition of the spectrum of the E_x_-component for sample B. When assuming the same LORE peak energy, intensity, and FWHM as the respective values of the A-type (2.0, 8.0) structure, the background emission component is plotted by the red dashed line. The spectral feature of the significant intensity reduction at 330 cm^−1^ in the E_x_-component is a clue to the dominant contribution of E_⊥_ polarization component (E_x_ for the type-A sample). The dotted line in Figure 6(b) shows the sum of 105% of the E_x_-component and 42% of the E_y_ component of the A-type (2.0, 8.0) sample, satisfying the intensity agreements at the LORE peak and 500 cm^−1^. The contribution of the E_y_-component of the type-A sample is lower than expected. This difference can be explained by the slightly narrower window width of the B-type sample. As shown in Figure 3(b), the blackbody-like emission significantly decreases with decreasing the window width; the decrease in *W*_W_ by 0.4 μm (2.0–1.6 μm) induces a reduction in the E_//_-blackbody-like emission at 500 cm^−1^ by 81% and the E_⊥_-component by 73%. A more significant decrease in the E_//_-component is exhibited via the narrowing of the window. In contrast, the variation in LORE peak intensity is 39%. These properties suggest that the narrower window width of the present B-type sample than that of the A-type (2.0, 8.0) sample by 0.2 μm can yield a lower intensity of the background with the polarization component parallel to the window line.

Here is a question about the function of the crossing area between the longitudinal (parallel to *y*) and lateral (parallel to *x*) window lines. As these two window lines mutually cross by a period of 10 μm in the square lattice, a 20% decrease in the LORE intensity is expected when considering these intersection regions with no facing metal stripe pairs in the x or y direction. We discuss the effects of these regions on the LORE intensity, accounting for several factors and constraints. When the LORE intensity is decreased by 20% from that of the A-type sample, the background spectrum is obtained as the dashed-and-dotted line in Figure 6(b). This function exhibits a double-peak structure in the region of 285–300 cm^−1^. The shoulder structure in the vicinity of the peak energy indicates the presence of the LORE component, which is invalid as a background spectrum. Next, when considering the 20% higher LORE intensity, the background spectrum (blue thin solid line) shows a local drop in emission intensity at 280 cm^−1^. This drop suggests the selective energy transfer to LORE. Because the background emission is independent of the electric dipole under consideration, a significant spectral drop at the emission peak energy is considered invalid. In the background spectrum of sample B shown in Figure 6(a), the fluctuation of the data points in the Reststrahlen band is negligible, indicating negligible intensity uncertainty. The uncertainty in the examined background spectra in Figure 6(b) also looks negligible. Thus, the spectrum drop and shoulder in the background emission in the LORE area are considered to be invalid. These discussions suggest that the LORE intensity in the E_x_-component is nearly identical to that of the sample A (2.0, 8.0) structure within the uncertainty of 20%. Now, we discuss the difference in *W*_W_ between the A-type (2.0, 8.0) sample and the B-type sample. The LORE intensity of the A-type (1.6, 8.4) structure was found to be 0.90 (±0.22) times the intensity of the (2.0, 8.0) structure. Assuming a linear dependence of the intensity on *W*_W_ in this range, the LORE intensity for the *W*_W_ of 1.8 μm is estimated to be 95 (±11) % of the *W*_W_ of 2.0 μm. The metal width *W*_M_ 10.8 μm is greater than 8.0 or 8.4 μm. According to our previous report summarizing various samples with various structural properties [19], the LORE intensity is nearly constant in the region currently under discussion, independent of the volume occupied by the electric flux connecting pairs of Au plates (supplemental material). We believe that the LORE of the A-type (2.0, 8.0) sample is suitable for the present analysis.

Thus, the LORE intensity for E_x_ of the B-type sample is thought to be almost the same as that of the A-type sample with the same *W*_W_, including an uncertainty of −15%. The uncertainty analysis allows an intensity, covering a slight increase, within +25%, but no rational reason is possible for the increase. Conclusively, the total LORE intensity of the square lattice structure, including E_x_ and E_y_ polarizations, is close to double the intensity of the A-type sample. A decrease of 15% is within the uncertainty range. When reducing *W*_W_ from 2.0 μm in B-type structure, the loss factor due to the intersection will decrease.

### 3.3. Concentric circular structure

The thermal emission spectra of the C-type (*W*_W_, *D*_M_) = (2.3, 7.6) and the A-type (2.0, 8.0) samples at 630 K are plotted in Figure 7(a). Here, the intensity of the A-type sample is normalized to that for the center circle length of the emission window of the C-type sample, which is 9.9π μm. The red-solid line and red-dashed line show the spectra for the E_x_ and E_y_ polarization components, respectively. This structure has 6-fold rotational symmetry, while the spectral difference between E_x_ and E_y_ polarization components is negligible. This result suggests that the difference in the distance of the ring structures, 7.8 μm along the *x*-axis and 22.6 μm along the *y*-axis, induces a negligible difference in the polarization charges and the electric dipole moment for LORE. The average LORE emission intensity of the E_x_ and E_y_ components of the C-type (2.3, 7.6) sample is higher than that of the A-type (2.0, 8.0) sample because the emission of the A-type sample is allowed only for the polarization perpendicular to the metal stripes.

**Figure 7.**
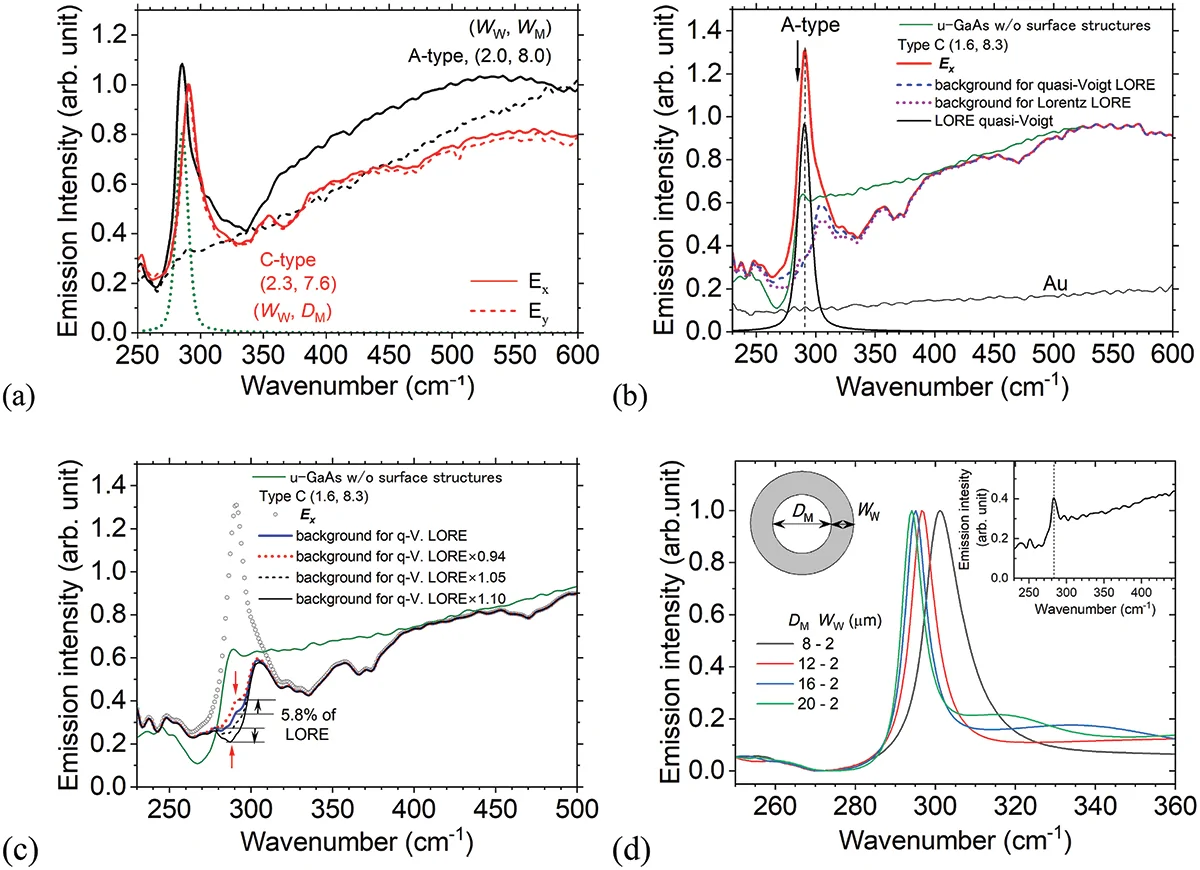
Emission spectra of type-C concentric circle structures, (a) the spectra for (*W*_W_, *D*_M_) = (2.3, 7.6) comparing with those of type a sample, (b) spectrum decomposition for the (*W*_W_, *D*_M_) = (1.6, 8.3) sample, taking the Lorentzian and quasi-Voigt function for the 100% LORE of the A-type (1.6, 8.4) sample and GaAs without metal component, (c) analysis of the LORE intensity using quasi-Voigt functions, and (d) peak energy shift calculated by the FDTD simulation. The inset of (d) is the experimental spectrum of C-type (2.0, 26) structure.

The spectrum of the C-type (2.3, 7.6) sample shows lower blackbody-like emission intensity at 500 cm^−1^ than that of the A-type (2.0, 8.0) sample. The window width in the *x*-direction of the C-type sample varies from 2.3 μm to 8.9 μm, and no longer. It is thought that the diffraction effect is more significant than in the A-type sample for the light polarized along the center circle, thereby suppressing background emission.

Figure 7(b) shows the spectrum of the C-type (1.6, 8.3), decomposed into the LORE and the background emission components. This sample has the same window width as the A-type (1.6, 8.4) sample. The LORE intensity and background spectrum of sample C were determined by considering the increase or decrease of the LORE intensity relative to that of the A-type (1.6, 8.4) structure. The background spectrum is obtained by subtracting the calculated LORE intensity of a trial function from the observed emission intensity, and the validity of the spectrum structure was investigated. As the spectrum peak energy shifted, as shown in Figure 7(b), the peak energy was adjusted to match the observed spectrum: 290.5 cm^−1^. The LORE function of the A-type (1.6, 8.4) structure was obtained by the least-squares fitting, as conducted for the A-type (2.0, 8.0) sample shown in Figure 3(b). We discuss two trial functions of quasi-Voigt and Lorentzian functions. Here, the following constraints on the background spectrum profile and intensity were applied. The upper limit of the spectrum intensity in the region of 300–350 cm^−1^ was set by the intensity criteria based on the bare GaAs sample, obtained using the same process as in Figure 6. This spectrum is shown as the green curve in this figure and indicates the emission intensity when SPhP formation is invalid. The second constraint is the background spectrum profile in the vicinity of 290 cm^−1^. When a high LORE intensity is assumed, the background intensity shows a significant spectrum drop at this radiation energy. However, the background intensity cannot be lower than the emission intensity of the Au film given by its surface coverage. Another factor is that the background emission spectrum has no significant dip at the LORE peak energy, as is the case with sample B.

When adopting the quasi-Voigt function, peak energy of 284.6 (±0.2) cm^−1^, FWHM of 12.8 (±0.8) cm^−1^, and *r*_L_ of 0.58 (±0.3) were obtained for the A-type (1.6, 8.4) sample. The background spectrum obtained by subtracting the LORE from the E_x_-component has the maximum uncertainty of ±2.9% of the LORE intensity in the Reststrahlen band (supplemental material). The background spectra for the 100% LORE of the A-type sample are plotted by the dashed line in Figure 7(b) and the solid line in Figure 7(c). The background functions of the C-type sample in a range of 94–110% of the LORE intensity of the A-type sample are plotted in Figure 7(c). The background spectrum for the 110% LORE of the A-type sample shows a dip at 288.3 cm^−1^, with a 5.8% decrease in intensity relative to the local maximum at 277.7 cm^−1^; a local spectrum dip is clearly observed. When the LORE intensity is 105% of the A-type sample, the spectrum is almost flat in the range of 277.7–288.3 cm^−1^. When the LORE intensity is 94%, the increase at 290 cm^−1^ from the background for the 100% LORE of the A-type sample is 5.8% of the 100% LORE intensity. At this energy, we can identify an additional peak as a spectral shoulder. Thus, the background spectra for the LORE peak intensity outside the range of 94–110% of the A-type sample exhibit an additional peak or a spectrum dip; these drops and peaks are considered to be invalid with respect to the uncertainty in the LORE intensity. Thus, we estimate that 94–110% of the A-type (1.6, 8.4) sample is the probable range of LORE intensity for the C-type sample.

When we adopt *r*_L_ = 1.0 (pure Lorentzian function), the peak energy of 284.8 (±0.1) cm^−1^, FWHM of 12.2 (±0.4) cm^−1^, and the integrated intensity of 1.17 (±0.03) times the quasi-Voigt function were obtained. The higher intensity than that of the quasi-Voigt function is probably due to the following fundamental property: when the FWHM and the peak intensity of a Lorentzian function are the same as those of a Gaussian, the spectrum-integrated intensity of the Lorentzian is higher than that of the Gaussian by 47.6%. The intensity coincidences of the two background profiles in the two areas, higher than 340 cm^−1^ and lower than 240 cm^−1^, and at the LORE peak energy, shown in Figure 7(b), support that analysis. The uncertainty of the background intensity in the Reststrahlen band is ±3.6% of the LORE intensity (supplemental material). The background function for the 100% LORE of the A-type sample is plotted by the dotted line in Figure 7(b). The probable range of radiation emission intensity was investigated, as in the case using the quasi-Voigt type LORE profile. When adopting a higher intensity of 11% of the A-type sample, an invalid spectrum dip was observed. It was found that the intensity at the local minimum, 287.3 cm^−1^, is lower than that at the local maximum, 261 cm^−1^, by 7% of the LORE intensity of the A-type sample. When 107% intensity of the A-type sample is adopted, the background spectrum was found to be nearly flat in this energy region. When the LORE intensity of 93% of the A-type sample, the invalid shoulder structure, indicating double peaks, is observed (supplemental material). When taking the Lorentzian, the intensity of the C-type (1.6, 8.3) sample is considered to be in a range of 93–115% of the A-type (1,6, 8.3) sample. This discussion reveals that the emission intensity of the C-type sample for a polarization component is nearly the same as the intensity of the E_x_-component of the A-type sample within the ambiguity of −7−+15%, suggesting that the total LORE efficiency of C-type samples is approximately double that of the A-type sample via the relaxation of polarization selection.

The FWHMs of 12.8 (±0.8) cm^−1^ and 12.2 (±0.4) cm^−1^ indicate THz emission lifetime of 0.415 and 0.435 ps, respectively. The background analysis indicates that the lifetimes of the 2-D structure are approximately equal. When considering the LO phonon decomposition lifetime of approximately 1ps, the radiative lifetime is estimated to be approximately 1ps (Supplemental material). When considering the inclusion of inhomogeneous broadening based on the quasi-Voigt property, the LO-phonon lifetime is estimated to be longer. The present results for the Lorentzian fraction of approximately 50% and the adaptability of the Lorentz function suggest that the lifetime can be of the same order.

The peak energy obtained from the spectrum fitting is 290.5 cm^−1^, which is higher than that of the A-type sample series (*W*_W_ = 0.6–10 µm). This feature is the same as that for the (2.3, 7.6) sample shown in Figure 7(a). Figure 7(d) shows the peak energy shift calculated by the FDTD simulation, where a broad Gaussian wave, peaked at 375 cm^−1^ and with an FWHM of 56 cm^−1^, is incident on a single-ring structure from the air side, and the emission spectrum was obtained by the Fourier transform. The LO phonon energy of 291 cm^−1^, accompanied by the physical constants at room temperature, is adopted. This figure shows that the peak approaches the LO phonon energy as the diameter increases. As this simulation was conducted on a single structure, the factor of the periodicity of the ring structures is excluded from the candidate reasons for the peak energy shift from the value for A-type samples. The periodic boundary condition for the circumference length of the window is excluded because the calculated peak energy variation is within 3%, even for the variation of 120% in the circumference length. It was found that the emission peak becomes broad and peak shifts to the higher energy side as the metal width decreases in the A-type structure: 283.4 cm^−1^ for (4.0, 10), 285.4 cm^−1^ for (4.0, 8.0), and 286.3 cm^−1^ for (4.0, 4.0) (Supplemental material). The interaction of the neighboring electric dipoles perturbs the formation of an electric dipole at the LO phonon energy. This effect is more significant for the ring structure. The spectrum of the C-type (2.0, 26.0) structure, shown in the inset of Figure 7(c), has a peak at 283.5 cm^−1^, while the LORE intensity is low due to the small fraction of the emission window area. The window width in the *x*-direction increases up to 15 μm, resulting in a lower intensity ratio of LORE to the background blackbody-like emission than those of (2.3, 7.6) and (1.6, 8.3) structures.

### 3.4. Two-dimensional periodicity effect

Figure 7(a,b) shows spectral dips at the energies of 370 cm^−1^, 470 cm^−1^, and 600 cm^−1^. The crossing points at 370 cm^−1^ and 600 cm^−1^ in the SPhP dispersion curve shown in Figure 4 are obtained when considering the periodic boundary condition along the circumference length of the emission window: 2π(*D*_M_+*W*_W_) and *p* = 2 and 3 in the SPhP condition shown in Figure 4. The periodicity of 20 μm along *x* cannot give the photon energy of the emission minima. The energy of another local minimum at 470 cm^−1^ is given for the periodicity of 12 μm for *p* = 1, corresponding to a periodicity of 60 μm and *p* = 5, which is the sum of the sides of the triangle formed by three centers of adjacent rings. Thus, the 2-D periodicity is substantialized in the spectral structure via the SPhP absorption features. In contrast, the LORE emission is robust in peak energy.

### 3.5. Rectangular emission window structure

The emission spectra for the D-type samples are shown in Figure 8. When (*W*_L_, *W*_s_) is (20, 2.0), the LORE peak is observed only for the E_x_-component, which is consistent with the result of the A-type samples. When *W*_L_ is decreased to 10 μm, the LORE peak is also observed for the E_y_ component, while its intensity is weaker than that of the E_x_-component of the (20, 2.0) structure. This property is attributed to the coherence of the LO-phonon between a pair of metal plates with a 10-μm distance or shorter, as shown for the A-type samples. In contrast, the LORE peak disappears when *L*_L_ is reduced to 6 μm. This *W*_L_ is short enough for the LO-phonon coherence. Figure 8(b) shows the reflectance spectra at 296 K for these samples. The reflectance drop at the LO phonon energy of 291 cm^−1^ at this temperature is observed only for the E_x_ polarization component in the A-type samples. In contrast, no absorption or emission is observed for the (6, 4) structure, indicating that the LO-phonon resonant absorption and emission are too weak to be observed or prohibited.

**Figure 8.**
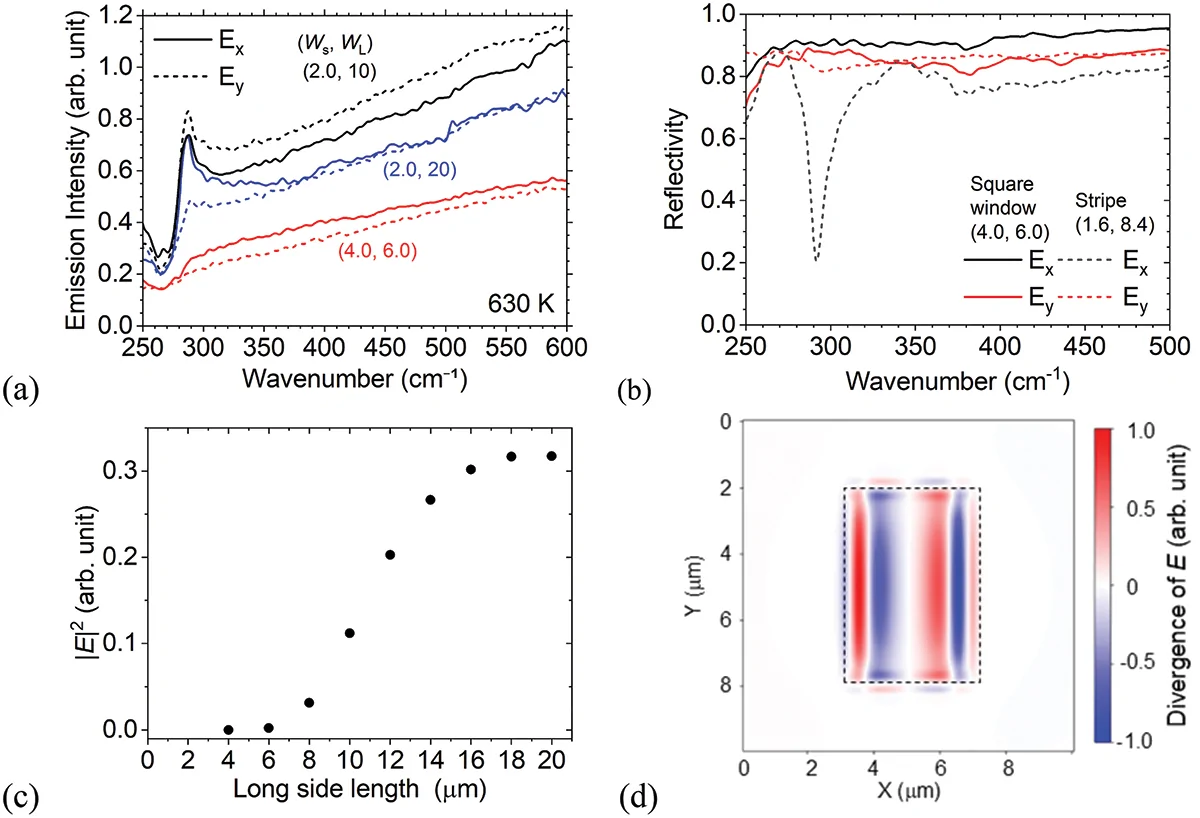
Emission properties of D-type rectangular window structures, (a) emission spectra at 630 K, (b) reflectance spectra at 300 K, (c) square of the electric field on the sample surface as a function of the longer side length, calculated by FDTD method, (d) images of the distributions of the divergence of electric field corresponding to the polarization charges calculated by FDTD method.

Figure 8 shows the emission intensity obtained by the FDTD simulation as a function of the longer-side length *W*_L_. In this simulation, the excitation light is incident on the sample from the air side. This intensity is calculated at the window surface at 0.476 ps after the incident light passes through the surface. This result shows that the LORE intensity decreases significantly as *W*_L_ decreases, consistent with the experimental result. The electric field in the rectangular window region is found to be four orders lower than that of the A-type structure. Figure 8 shows the divergence of the electric field at the surface, indicating no formation of electric dipoles. Thus, the forbidden formation of the electric dipole is the origin of the non-observation of LORE. When considering two pairs of coherently vibrating positive and negative polarization charges placed on the opposite sides or corners of the rectangular window, the formation of a quadrupole is considered. In this case, the dipole component is highly reduced. It is considered that the condition of *L*_L_ > *L*_S_ induces the dominance of stripe structures of the divergence of the electric field *E* along the longer side.

The Hamiltonian of the electric dipole emission is represented by the formula of $q\Sigma r_{j}E(0)$. In contrast, $q\Sigma (r_{j}\;\cdot \;\nabla )\;(r_{j}\;\cdot \;E(0))/2$, is the expression for quadrupole, where *E* is the electric field [31]. By approximating $\nabla E\left(r\right)$ as ω*E*(0)/*c*, the magnitude of the Hamiltonian of the quadrupole is calculated to be 1.7% of that of the dipole. Thus, the emission is considered to be too weak to be detected.

We have observed that line-and-space structures of high-electron-density regions with a density of 1 × 10^20^ cm^−3^ behave as metallic regions and yield LORE using a GaN wafer [20] . These results indicate that it is possible to control the emission and extinction of LORE by optical excitation or current injection into stripe regions, yielding electron density regions.

## 4. Conclusion

We have investigated thermal radiation resonant with the LO phonon from 2-D surface metal (Au) structures with square lattices and ring structures on u-GaAs, comparing the results with those from 1-D line-and-space structures. 75-nm-thick Au films were deposited on GaAs wafers using photolithography and sputtering. Polarization-resolved radiation emission spectra were observed at 630 K and 323 K using an FTIR spectrometer. Emission peaks at 285–292 cm^−1^ and FWHM of 11–13 cm^−1^ were observed. Although the LORE emission from the line-and-space structures was observed only for polarization perpendicular to the stripe structures, the 2-D emission window structures of square lattices and rings exhibit LORE in both x- and y-polarizations. This relaxation of polarization selection yielded the LORE of approximately double the intensity of that of the 1-D line-and-space structure. Although the photon energies of the observed spectrum minima due to optical absorption via SPhP depend on the periodicity of surface structures, the LORE is robust in its emission peak energy. A small deviation from the LO-phonon energy within 5 cm^−1^ was observed for a ring structure, which was attributed to the insufficient formation of polarization charges at Au/GaAs interfaces. When we provide sufficient metal area to form sufficient interface polarization charges, the radiation emission intensity is dominated by the total length of the stripes, independent of the 2-D stripe structure (e.g. ring or square lattice). In contrast, the structure of a rectangular square emission window with a size less than the LO-phonon coherence length gives no LORE, indicating the forbidden formation of the electric dipole. These 2-D metal surface structures within the LO-phonon coherence length enable versatile control of the emission and prohibition of continuous THz waves [32].

## Supplementary Material

Supplemental Material
