# Supplementary material for: Emission control of longitudinal optical phonon resonant thermal terahertz radiation from two-dimensional surface metal micro-structures on GaAs
Source: Sci Technol Adv Mater. 2026 Sep 21;27(1):2724893. doi: 10.1080/14686996.2026.2724893 (PMC13715342; doi:10.1080/14686996.2026.2724893)
Supplement: Supplementary file 1 [file TSTA_A_2724893_SM0778.docx]

Supplemental material

Emission control of longitudinal optical phonon resonant thermal terahertz radiation from two-dimensional surface metal micro-structures on GaAs

Takumi Ohshima, Daiki Yoshikawa, and ^*^Yoshihiro Ishitani

*Graduate School of Science and Engineering, Chiba University*

*1-33 Yayoicho, Inage-ku, Chiba, 263-8522, Japan*

^*^e-mail) ishitani@faculty.chiba-u.jp

A. Background emission spectra of the C-type (1.6, 8.3) structure

Figure S1 shows the peak fitting of the radiation emission spectrum of the A-type (1.6, 8.4) sample and the background emission spectra of the C-type (1.6, 8.3) structure. Open circles in Figure S1a show the experimental spectrum of E_x_ – E_y_. The fitting was conducted in the range of 250–290 cm^-1^. The background spectra of the type-C (1.6, 8.3) sample were obtained by subtracting the LORE intensity from the E_x_-component of radiation. Two functions of quasi-Voigt and the Lorentzian were adopted. Adjusted parameters for the A-type (1.6, 8.4) samples are peak: 284.6(±0.2) cm^-1^, FWHM: 12.8(±0.8) cm^-1^, *r*_L_: 0.58(±0.3), intensity: 3.82 (±0.18) for quasi-Voigt function and 284.8(±0.1) cm^-1^, FWHM: 12.2(±0.4) cm^-1^, intensity: 4.46 (±0.12) for Lorentzian. The background spectra show oscillations in the Reststrahlen band. The peak intensity ratio of the Lorentzian against the quasi-Voigt function is 1.17. The peak at 310 cm^-1^ is attributed to the over-purging of the optical path in the sample measurement.

In the analysis of the C-type (1.6, 8.3) structure, the peak energy was adjusted to the experimental data of 290.5 cm^-1^. Figure S1c shows the background analysis for the Lorentzian LORE: 95%, 107 %, and 115% of the LORE of the A-type sample. Invalid double-peak feature is observed for 95% LORE, and invalid spectrum dip is observed for 115% LORE.

(a) (b)

(c)

Figure S1 Background emission spectra for various LORE peaks, (a) ambiguity of quasi-Voigt-type LORE intensity, (b) spectrum separation of LORE and the background emissions for type-A (1.6, 8.4) structure, and (c) ambiguity of the Lorentzian LORE intensity for sample C (1.6, 8.3) structure.

q-V. : quasi-Voigt function, L.: Lorentz function.

B. Emission probability

The LO-phonon resonant radiation emission rate *K* is expressed using the Bose-Einstein occupation factor of the LO phonon in Equation (S1).

*K* = $\frac{\pi}{2\hbar}\left( \hbar\omega\right)^{2}\frac{\left( SW \right)^{2}}{V_{\omega}V_{L}}{C\varepsilon}_{0}\left( \frac{1}{\varepsilon\left( \infty\right)}-\frac{1}{\varepsilon\left( 0 \right)} \right)\delta\left( \hbar\omega-\hbar\omega_{L} \right)\left[ \frac{1}{\exp\left( \hbar\omega_{L}{/k}_{b}T \right)-1} \right]d\nu$ , (S1)

where *V*_ω_ and *V_L_* are the volumes covered by a photon and an LO phonon, respectively. dν is the associated number of states, respectively. *W* is the effective window width. *S* is the area with the polarization charges. *C* is the factor representing the generation efficiency of the polarization charge, and *S* is the effective area covered by the polarization charges. By using Equation (S1), we obtain the radiative lifetime *K*^-1^ ≈50⋅*V*_ω_ *V_L_* /(*Wd*)^2^ in the unit of fs. In addition, *V*_L_ is estimated to be equal to (*SW*). Thus, the radiative emission efficiency depends on the ratio of the volume of the photon coherence and that of the phonon in the formula of *K*^-1^ ≈50⋅*V*_ω_ /*V_L_* (fs). The present lifetime values estimated from the FWHM values of LORE, 11-13 cm^-1^ give the lifetime of 470-410 fs. When considering the LO phonon coherence of one window width, 2 μm, and radiation coherence as the wavelength, giving *V*_ω_ /*V_L_* as approximately 18, the radiative lifetime is estimated to be 450 fs.

Our previous analysis by comparing the LORE intensity with the thermal emission intensity of a pure graphite sample at 630 K gives a radiative lifetime of 1 ps. When considering an estimated LO phonon lifetime of 1 ps, which is obtained by extrapolating a reference data [S1], the radiative lifetime is estimated to be 1 ps, which is interpreted as a nonradiative LO phonon lifetime. By considering radiative and nonradiative lifetimes, the LO phonon lifetime in the present structures is obtained as 0.5 ps, which satisfactorily agrees with the lifetime obtained from the FWHM of 11-13 cm^-1^. Thus, the LORE intensity estimation for 2-D structures is considered valid.

C. Dependence of emission intensity on the LO-phonon related volume

Figure S2 shows the dependence of the LORE intensity on the volume occupied by the electric flux connecting pairs of Au plates (*V*_C_). *V*_C_ is expressed as (*W*_W_+*W*_M_)×*D*. *D* is the penetration depth of the electric flux starting from a metal plate, obtained using the results of the FDTD simulation. *D* of 3 μm is considered appropriate [18, 19]. Here, this volume is for 1μm depth perpendicular to the plane of this figure. Figure S2a shows the geometrical image of *V*_C_. LORE intensity is plotted as a function of for various samples, including mesa structures. For mesa structures, the mesa volume is added to *V*_C_. The emission intensity is plotted in Figure S2b. The plots without notation are for mesa structures. Emission intensity depends on *V*_C_, as well as on both *W*_W_ and *W*_M_. For the B-type sample, the stripe structure (*W*_W_, *W*_M_) = (1.8, 10.2) has a *V*_C_ of 36 µm^3^. At this volume, the emission intensity of no-mesa structures saturates and remains nearly constant. In analyzing the B-type sample, given the wide metal width of 10.8 μm and a narrow window width of 1.8 μm, the emission intensity of the A-type (2.0, 8.0) is suitable for use.

(a) (b)

Figure S2	LORE intensity as a function of the volume occupied by the electric flux related to the LO-phonon resonant electric dipole, (a) cross-sectional image of the sample structure with the definition of volume of electric flux connecting pairs of metal plates, *V*_C_, and (b) LORE intensity per line as a function of *V*_C_.

D. Variation of peak energy of LORE

Figure S2 Dependence of thermal emission spectra at 630 K on metal width. The emission window width is constant as 4.0 μm.

Figure S3 shows the emission spectra for A-type (4.0, 4.0), (4.0, 8.0), and (4.0, 10.0) structures. As the metal width decreases, the peak energy increases: 283.4 cm^-1^ for (4.0, 10), 285.4 cm^-1^ for (4.0, 8.0), and 286.3 cm^-1^ for (4.0, 4.0). The spectra show broadening toward higher energy. Figure 1a suggests that the positive and negative polarization charges can cancel when the metal width is not large enough to separate them, leaving an incomplete electric dipole. When the dipole formation is incomplete, the emission peak shifts to higher energy side for the structures of GaN ^20^. The increase in emission intensity at higher energies than the peak suggests a transition to the bare GaAs wafer spectrum as the metal width decreases.

Reference

(S1) Vallee F. Time-resolved investigation of coherent LO-phonon relaxation in III-V semiconductors. Phys. Rev. B 1994; 49, 2460-2468
